# Association of emotional support with quality of life, mental health, and survival in older adults with gastrointestinal malignancies–Results from the CARE registry

**DOI:** 10.1002/cam4.6477

**Published:** 2023-08-30

**Authors:** Daniel Clausing, Mackenzie E. Fowler, Christian Harmon, Abigail Tucker, Darryl Outlaw, Mehmet Akce, Bassel El‐Rayes, Smith Giri, Grant R. Williams

**Affiliations:** ^1^ Department of Medicine University of Alabama at Birmingham Birmingham Alabama USA; ^2^ Institute for Cancer Outcomes & Survivorship, University of Alabama at Birmingham Birmingham Alabama USA; ^3^ O'Neal Comprehensive Cancer Center University of Alabama at Birmingham Birmingham Alabama USA

**Keywords:** aging, cancer, emotional support, geriatric oncology, health‐related quality of life

## Abstract

**Background:**

Emotional support (ES) is the most frequently reported support need among older adults with cancer. Yet, the association of ES with cancer outcomes is largely unknown. This study examined the association of ES with health‐related quality of life (HRQoL), mental health, and survival among older adults with gastrointestinal (GI) malignancies.

**Methods:**

We included newly diagnosed older adults (≥60 years) with GI cancer undergoing self‐reported geriatric assessment at their first clinic visit. ES was measured using an adaptation of the Medical Outcomes Study (dichotomized adequate ES vs. inadequate ES). Outcomes included physical and mental HRQoL, anxiety, depression, and survival. Multivariable linear regression evaluated the association between ES and HRQoL scores. Multivariable logistic regression evaluated the association of ES with anxiety and depression. All models were adjusted for age at geriatric assessments, race, sex, and cancer type/stage.

**Results:**

795 participants were included. Median patient age was 68 years (IQR: 64–74), 58% were male, and most cancers were either colorectal (37.9%) or pancreatic (30.8%). Most (77.6%) had adequate ES. Patients with inadequate ES were more likely to be Black (31.5 vs. 20.8%, *p* = 0.005), disabled (24.1 vs. 10.4%, *p* < 0.001), widowed/divorced (54.2 vs. 24.8%, *p* < 0.001) and had lower physical and mental HRQoL t‐scores (Physical *β*: −3.35, 95% CI: −5.25, −1.46; Mental *β*: ‐2.46, 95% CI: −4.11, −0.81) and higher odds of depression (aOR: 2.22, CI: 1.34–3.69). This study found no difference between those with adequate ES versus inadequate ES in the proportion of deaths within 1 year of diagnosis (24.3% vs. 24.2%, *p* = 0.966), or within 2 years of diagnosis (32.4% vs. 33.2%, *p* = 0.126).

**Conclusions:**

Older adults with inadequate ES have worse physical and mental HRQoL and higher odds of depression compared to those with adequate ES.

## 1 INTRODUCTION

Older adults with cancer are more likely to experience depression, anxiety, and poor quality of life than those without cancer.^1^, ^2^ In fact, nearly 40% of older adults with cancer have clinically significant anxiety and reduced quality of life.^3^ Furthermore, the difference in psychological well‐being and the quality of life of cancer patients and their healthy peers increases with age.^4^ Inversely, older patients with cancer are *more* likely to prioritize quality of life considerations when making cancer treatment decisions than their younger counterparts.^5^ A patient's perception of their symptoms and how their cancer treatment might affect the quality of their life has been previously defined elsewhere in the oncology literature as health‐related quality of life (HRQoL).^6^

An increasingly recognized component of HRQoL in older adults with cancer is social support.^7^, ^8^, ^9^ Although the association between HRQoL and the broad construct of social support has been previously demonstrated, the relative contributions of different domains of social support (emotional support, informational, instrumental, etc.) to HRQoL has not yet been clarified. While definitions of social support and its subcomponents vary; emotional support is nearly always a separate and distinct component.^10^, ^11^ Emotional support has previously been defined implicitly as the collection of individual items such as “do you have someone to have fun with?” and “do you have someone to share your worries with?”, etc.^12^ If one were to synthesize these individual items into a single definition, emotional support would be defined as the perception that one has an intrapersonal relationship (or relationships) within which they can share emotions and be understood. One study of unmet social support needs reported that emotional support was the most common need among older adults with cancer (49.5%).^13^ A longitudinal social support study in patients with breast cancer suggested that emotional support is more closely linked to improved HRQoL after diagnosis than other domains of social support.^8^ Similarly, an article by Lapinsky et al reviewed the literature on HRQoL in older patients with colorectal cancer and concluded that more research is needed to understand the factors that “may have both a positive and a deleterious effect on HRQoL”.^14^ Another study in colorectal cancer patients of all ages showed that adequate social support was closely associated with improvements in depression and anxiety after 1 year.^9^ However, few studies to date have specifically investigated the relationship between emotional support and HRQoL in older adults with cancer.

In order to better understand the role of emotional support, we sought to examine emotional support and its association with HRQoL, mental health, and survival among older adults with gastrointestinal (GI) malignancies.

## 2 METHODS

### 2.1 Study population

Participants in the current study were selected from the Cancer & Aging Resilience Evaluation (CARE) registry. The CARE registry is an ongoing prospective registry of older adults (≥60 years of age) with cancer that has been collected since 2017.^15^ The CARE registry combines self‐reported geriatric assessments (GA) (entitled the CARE tool) and follow‐up outcomes. The CARE tool collects information from older patients across 10 domains: functional status, physical function, HRQoL, nutrition, social/emotional support, psychological status, cognitive function, comorbidities, social activities, and social determinants of health. The tool was modeled after the Cancer and Aging Research Group (CARG) GA developed by Arti Hurria with changes made to allow for completely patient‐reported assessment and to exchange certain portions of the cognitive and mental health assessments with validated tools that were easier to implement and score.^15^, ^16^, ^17^ Patients were prospectively enrolled in the CARE registry at their initial visit to the medical oncology clinic at the University of Alabama at Birmingham (UAB) after obtaining informed consent. Although the age cutoff for using the GA among adults with cancer is uncertain, 60 years of age was chosen as the first year of participation because prior research has shown similar rates of GA‐identified impairments across older age groups (ages 60–64, 65–74, and ≥ 75 years).^18^ For the current analysis, participants were excluded if: (1) GA completed more than 3 months prior to or more than 6 months after date of cancer diagnosis and/or (2) diagnosed with a non‐GI malignancy. This study was approved by the Institutional Review Board of the UAB (IRB‐300000092) and was performed in accordance with the ethical standards of the 1964 Declaration of Helsinki and later amendments.

### 2.2 Emotional support

Self‐reported emotional support was the primary exposure of interest. The CARE tool includes four items from the previously validated Medical Outcomes Study (MOS) social support survey to assess emotional support.^10^, ^12^, ^13^ Previous work has demonstrated strong psychometric performance in the assessment of social support using only two of the original MOS subsections (emotional and tangible support) termed the modified medical outcomes study social support survey (mMOS‐SS). This modified survey uses four items assessing emotional support and four items assessing tangible support and these were included in the CARE tool.^12^ Only the four emotional support items were included for analysis in accordance with the purpose of this study. Individual items include “do you have someone to have a good time with?”, “do you have someone to turn to for suggestions about how to deal with a personal problem?”, “do you have someone who understands your problems?”, and finally “do you have someone to love and make you feel wanted?”. Participants were instructed to indicate whether they had support *None of the time* (1 point), *A little of the time* (2 points), *Some of the time* (3 points), *Most of the time* (4 points), or *All of the time* (5 points). Responses were scored on a Likert scale, ranging from 1 to 5. Participants were considered to have adequate emotional support if they felt supported *most* or *all of the time* (Likert score ≥4) for every question. If a participant reported that they had emotional support *some of the time* or less frequently (Likert score ≤3) on ANY item they were considered to have inadequate emotional support, based on the prior literature.^13^

### 2.3 Primary outcome: Health‐related quality of life

The primary outcome of interest was HRQoL, measured using the National Institutes of Health Patient‐Reported Outcomes Measurement Information System® (PROMIS) 10‐item global assessment. The CARE tool includes the PROMIS® scale, which includes physical and mental health subscales scored as t‐scores.^19^, ^20^ Previous studies of the general US population have produced a standardized mean score of 50 with a standard deviation of 10.^19^ For the current study, we evaluate t‐scores on the physical and mental health subscales as continuous variables.^21^

### 2.4 Secondary outcome: Mental health

The secondary outcome of interest was mental health, specifically anxiety, or depression. Depression was assessed in the CARE tool utilizing the PROMIS® Depression Short Form 4a v1.0 which asks how often in the past week (*Never*, *Rarely*, *Sometimes*, *Often*, or *Always*) the patient has experienced depressive symptoms of worthlessness, helplessness, depression, or hopelessness in four separate questions.^22^, ^23^, ^24^, ^25^ The Short Form 4a v1.0 has previously been validated in older patients and in patients with cancer.^26^, ^27^, ^28^ Responses were converted to t‐scores and dichotomized with t‐scores ≥60 versus t‐scores <60 indicating the presence versus absence of clinically significant depression, respectively, in accordance with prior work demonstrating that t‐scores >60 (moderate–severe depression) are associated with clinically significant poorer outcomes (falls, performance status, pain, fatigue, dependence in IADL's/ADL's, anxiety, etc.).^29^, ^30^

The anxiety assessment in the CARE‐GA is the PROMIS® Anxiety Short Form 4a v 1.0 which has been previously validated in patients with cancer and used in prior CARE tool studies.^31^, ^32^ This assessment asks participants to answer how often in the past 7 days (Never, Rarely, Sometimes, Often, or Always) did the patient experience anxiety symptoms of fearfulness, difficulty focusing on anything other than anxiety, overwhelmed by emotions, or uneasiness across 4 questions. Responses were scored according to the PROMIS® Anxiety Short Form 4a v. 1.0. Patients with t‐scores ≥60 versus <60 indicating presence versus absence of clinically significant anxiety (labeled moderate–severe anxiety by PROMIS®).^22^, ^33^

### 2.5 Survival

Vital status up to May 15, 2022 was obtained by linking the cohort to the Accurint database,^34^ which contains death information from the Social Security Administration, obituaries, and state death records. We supplemented these data with medical record information. We calculated follow‐up time from date of diagnosis in months. We set the last date of follow‐up as May 15, 2022 corresponding to the last date of data linkage to the Accurint data or as date of death, whichever occurred first. To set up appropriate survival analyses (see Statistical Analysis section below), we classified 1‐year, 2‐year, and overall survival from date of diagnosis. For 1‐year survival, we censored participants at 12 months if no death had occurred as of the last date of follow‐up or if death occurred greater than 12 months after diagnosis. For 2‐year survival, a similar strategy was used with 24 months as the cut‐point. For overall survival, participants were censored at the last date of follow‐up if death had not yet occurred.

### 2.6 Covariates

Age at GA completion, race, ethnicity, education, employment, and marital status were obtained by self‐report as part of the CARE tool. Cancer type and cancer stage were extracted through individual chart review from the electronic medical record.

### 2.7 Statistical analyses

Differences in demographics and GA outcomes were assessed between patients with inadequate versus adequate emotional support status using chi‐square tests and *t*‐tests for categorical and continuous variables, respectively. Fisher's exact tests were used where expected cell size was low for categorical variables and the Mann–Whitney U test was used for non‐normal continuous variables. Linear regression was used to evaluate the association between emotional support status and HRQoL t‐scores. Logistic regression was used to evaluate the association between emotional support status and mental health outcomes. Both unadjusted and adjusted models were built; the latter included additional variables: age, race, sex, cancer type, and cancer stage as covariates based on the existing literature. Finally, Kaplan–Meier curves and log‐rank tests were used to evaluate the association between emotional support status and 1‐year, 2‐year, and overall survival. The proportional hazards assumptions were violated for survival outcomes and emotional support status so regression models of survival were not performed. Statistical significance was set at *α* = 0.05 and all analyses were performed using SAS Version 9.4.^35^

## 3 RESULTS

**FIGURE 1:**
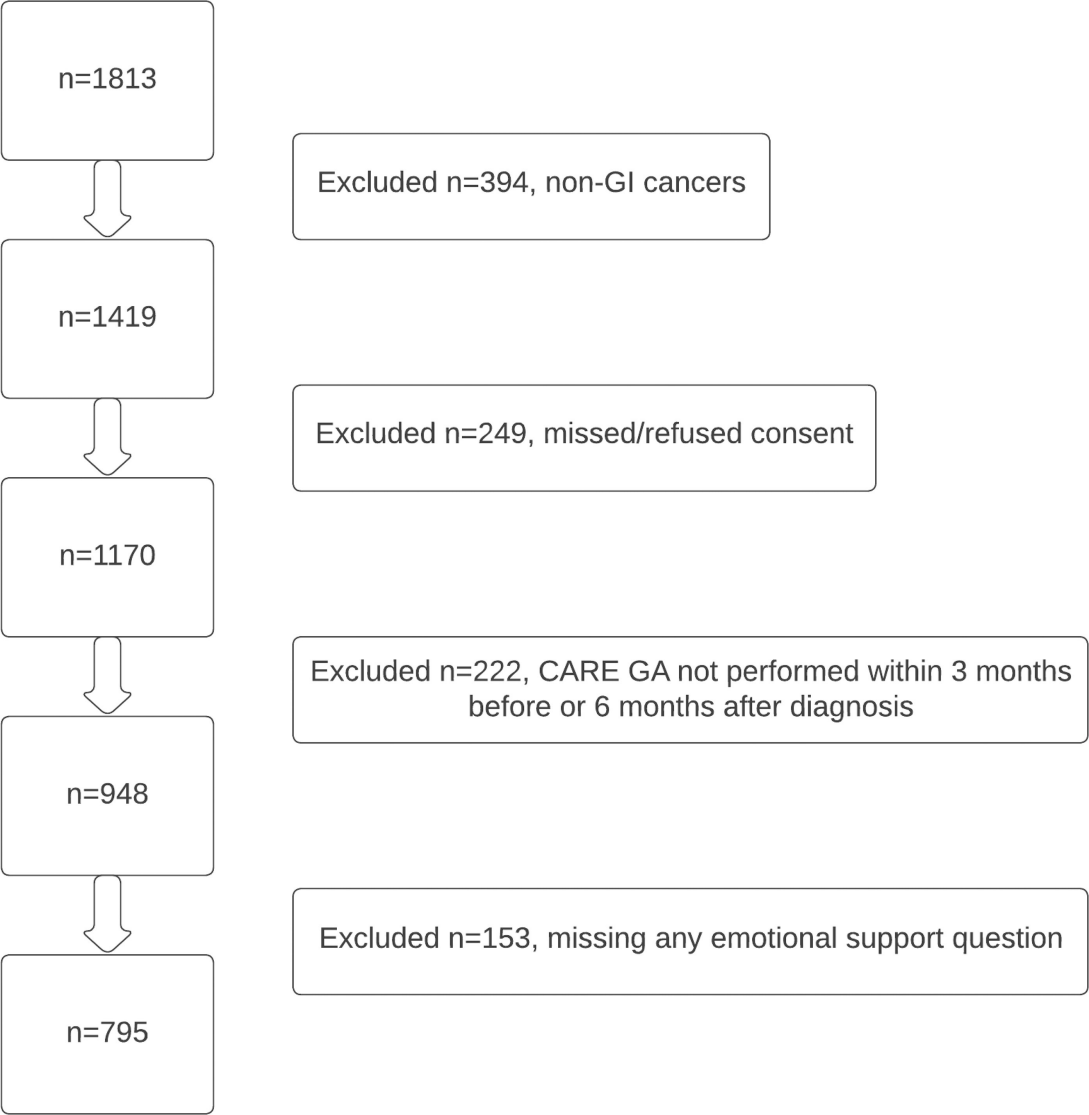
Consort diagram of study cohort.

Of 1813 participants within the CARE registry, 1419 had GI malignancies, 1170 consented, 948 completed the GA between 3 months prior to and 6 months after cancer diagnosis, and 795 had no missing emotional support data (Figure 1). Median age of the final analytic sample was 68.0 years (IQR: 64.0, 75.0). The study sample was 58.0% male, 74.6% White, and 97.5% non‐Hispanic (Table 1). A majority were also married (61.9%) and retired (61.2%). The most common malignancies included colorectal (37.9%, *n* = 274) and pancreatic cancer (30.8%, *n* = 223). Malignancies were advanced at enrollment with 72.1% (*n* = 564) diagnosed with Stage III or IV disease. Overall, 36.7% (*n* = 292) of participants died during the study period, 24.3% within 1 year of diagnosis, and 32.6% within 2 years of diagnosis (Table 1).

**TABLE 1:** Participant Characteristics at Baseline by Emotional Support.

Variable	Total	Inadequate Emotional Support 178 (22.4%)	Adequate Emotional Support 617 (77.6%)	*p*‐Value*
Age, median (IQR)	69.6 (7.6)	69.8 (7.9)	69.6 (7.5)	0.768
Sex, male, *n* (%)	461 (58.0)	97 (54.5)	364 (59.0)	0.284
Race, *n* (%)				**0.005**
White	593 (74.6)	116 (65.2)	477 (77.3)	
Black	183 (23.1)	56 (31.5)	128 (20.8)	
Other/unknown	18 (2.3)	6 (3.4)	12 (1.9)	
Ethnicity, *n* (%)				0.337
Hispanic/Latino	18 (2.3)	5 (2.8)	13 (2.1)	
Non‐Hispanic/Latino	775 (97.5)	172 (96.6)	603 (97.7)	
Other/unknown	2 (0.3)	1 (0.6)	1 (0.2)	
Marital status, *n* (%)				**<0.001**
Single	53 (6.9)	22 (13.1)	31 (5.2)	
Married	474 (61.9)	55 (32.7)	419 (70.1)	
Widowed/divorced/separated	239 (31.2)	91 (54.2)	148 (24.8)	
Employment, *n* (%)				**<0.001**
Disabled	100 (13.2)	40 (24.1)	60 (10.1)	
Retired	465 (61.2)	93 (56.0)	372 (62.6)	
Part‐time ≤ 32 h/week	26 (3.4)	5 (3.0)	21 (3.5)	
Full‐time > 32 h/ week	92 (12.1)	14 (8.4)	78 (13.1)	
Other	77 (10.1)	14 (8.4)	63 (10.6)	
Education, *n* (%)				0.144
Did not graduate high school	102 (13.3)	30 (17.8)	72 (12.1)	
High school graduate	370 (48.3)	84 (49.7)	286 (47.9)	
Completed college degree (Associate's/Bachelor's)	206 (26.9)	40 (23.7)	166 (27.8)	
Advanced degree	88 (11.5)	15 (8.9)	73 (12.2)	
Gastrointestinal malignancy, *n* (%)				0.163
Colorectal	274 (37.9)	72 (45.9)	202 (35.7)	
Pancreatic	223 (30.8)	39 (24.8)	184 (32.5)	
Hepatobiliary	56 (7.8)	11 (7.0)	45 (8.0)	
Esophageal‐gastric	73 (10.1)	17 (10.8)	56 (9.9)	
Other	97 (13.4)	18 (11.5)	79 (14.0)	
Cancer stage, *n* (%)				0.234
Stage I/II	218 (27.9)	55 (31.4)	163 (26.9)	
Stage III/IV	564 (72.1)	120 (68.6)	444 (73.2)	
Death during Study period, *n* (%)	292 (36.7)	55 (37.1)	226 (36.6)	0.913
Overall follow‐up time, mean (SD)	19.8 (14.9)	18.5 (14.7)	20.1 (15.0)	
Death 1 year post‐diagnosis, *n* (%)	193 (24.3)	43 (24.2)	150 (24.3)	0.966
One‐year mean follow‐up time, mean (SD)	9.8 (3.2)	9.7 (3.3)	9.9 (3.2)	0.452
Death 2 years post‐diagnosis, *n* (%)	259 (32.6)	59 (33.2)	200 (32.4)	0.855
Two‐year mean follow‐up time, mean (SD)	14.9 (7.9)	14.2 (7.7)	15.2 (7.9)	0.126

* Estimated using Mann–Whitney *U* for age, *t*‐tests for other continuous variables, and chi‐squared tests for categorical variables.

Values in bold indicate a statistically significant variable with *p*‐value <0.05.

**FIGURE 2:**
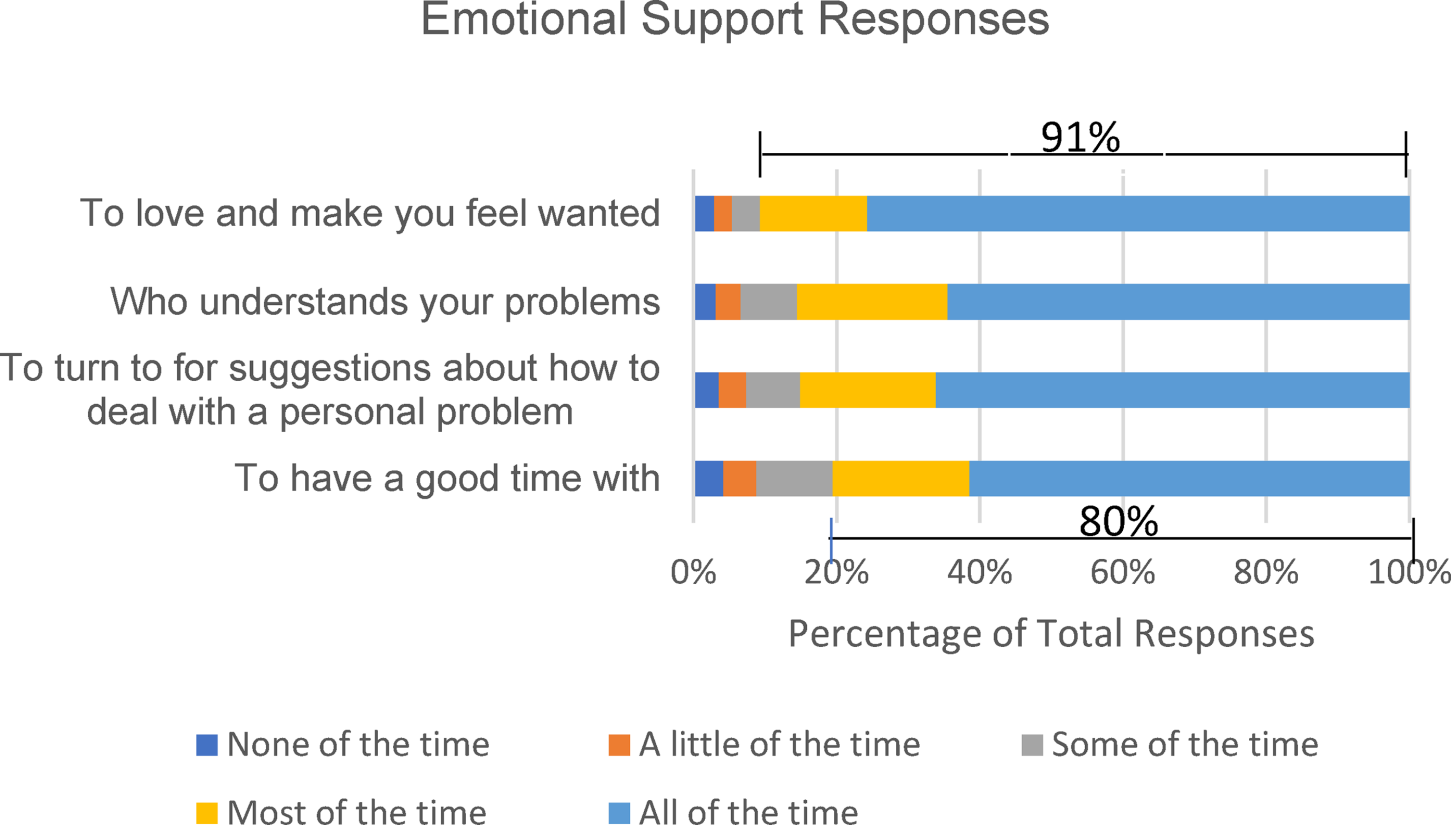
Frequency of particular responses to emotional support questions.

More than three‐quarters (77.6%) had adequate emotional support (Table 1). More than 80% indicated they had each type of emotional support (e.g., someone to have a good time with, someone who understands your problems, etc.) *most* or *all of the time* (Figure 2). Patients most frequently reported adequate emotional support for the question “do you have someone to love and make you feel wanted?” with 91% receiving that type of support *most of the time* or more often. Patients least frequently reported adequate emotional support for the question “do you have someone to have a good time with?” with 80% of patients receiving that type of support *most of the time* or more often. Patients with inadequate emotional support were more likely than those with adequate emotional support to be Black (31.5% vs. 20.8%, *p* = 0.005) or another race (3.4% vs. 1.9% *p* = 0.005) but less likely to be White (65.2% vs. 77.3%, *p* = 0.005) (Table 1). Patients with inadequate emotional support were more likely to have a disability (24.1% vs. 10.4%, *p* < 0.001) and less likely to be retired (56.0% vs. 62.6%, p < 0.001), employed part‐time (3.0% vs. 3.5%, *p* < 0.001) employed full time (8.4% vs. 13.1%, *p* < 0.001), or to have selected other for employment (8.4% vs. 10.6%, *p* < 0.001). Patients with inadequate emotional support were more likely to be widowed/divorced/separated (54.2% vs. 24.8%, *p* < 0.001) or single (13.1 vs. 5.2, *p* < 0.001) and less likely to be married (32.7% vs. 70.1%, *p* < 0.001).

After adjustment for age, race, sex, cancer type, and cancer stage, inadequate emotional support was associated with a 3.35 point lower t‐score for physical HRQoL (*β*: −3.35, 95% confidence interval (95% CI): −5.25, −1.46) and a 2.46 point lower t‐score for mental HRQoL (*β*: ‐2.46, 95% CI: −4.11, −0.81) (Table 2). Inadequate emotional support was also associated with 2.2‐fold higher odds of depression (aOR: 2.22, CI: 1.34, 3.69), No statistically significant association was observed between inadequate emotional support and anxiety (aOR: 1.15, 95% CI: 0.73, 1.81) **(**Table 2 **)**.

**TABLE 2:** Association between Low Emotional Support and health‐related quality of Life (HRQoL), depression, and anxiety.

Variable	Unadjusted estimate (95% CI)	Adjusted estimate (95% CI)^a^
Physical HRQoL t‐score, *β* ^b^	−2.86 (−4.67, −1.06)	−3.35 (−5.25, −1.46)
Mental HRQoL t‐score, *β*	−2.92 (−4.46, −1.38)	−2.46 (−4.11, −0.81)
Anxiety, OR^c^	1.22 (0.81, 1.84)	1.15 (0.73, 1.81)
Depression, OR	2.14 (1.36, 3.37)	2.22 (1.34, 3.69)

^a^ Adjusted for age, race, sex, cancer type, and cancer stage.

^b^ Estimated using linear regression.

^c^ OR = odds ratio; estimated using logistic regression.

**FIGURE 3:**
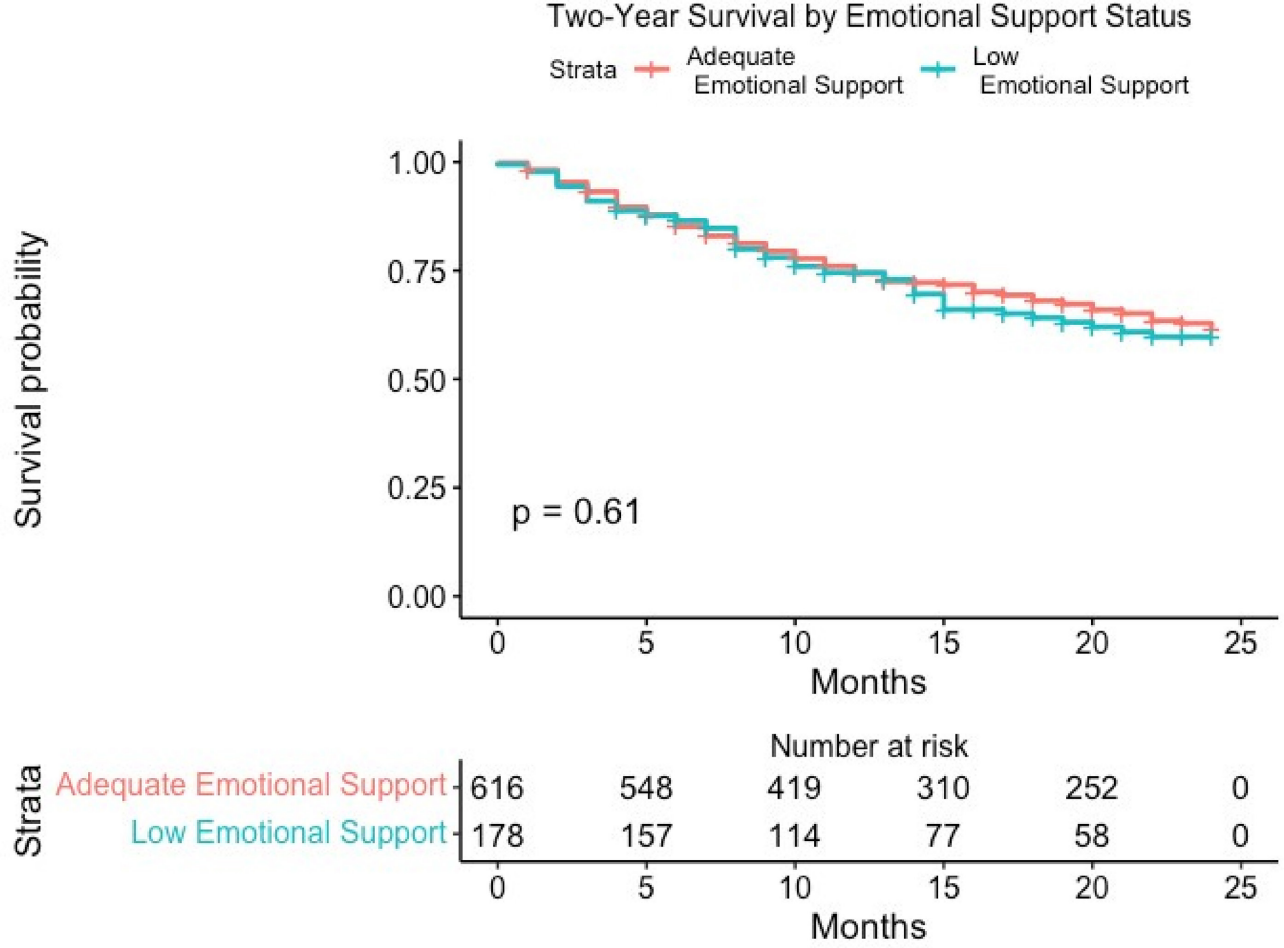
Two‐year Kaplan–Meier survival curve comparison with adequate versus low emotional support.

Median follow‐up time in during the study period was 14 months (IQR: 8–31 months). Overall, there were 292 (32.6%) deaths during the study period. There were no differences between patients with adequate emotional support and those with inadequate emotional support in the proportion of deaths during the study period (36.6% vs. 37.1%, *p* = 0.913), within 1 year of diagnosis (24.3% vs. 24.2%, *p* = 0.966), or within 2 years of diagnosis (32.4% vs. 33.2%, *p* = 0.126). There was no significant difference in survival probability between patients who had adequate emotional support versus those with inadequate emotional support (1‐year survival: *p* = 0.940; 2‐year survival: *p* = 0.544; overall survival: *p* = 0.506) (Figure 3).

## 4 DISCUSSION

In this cross‐sectional study of older adults with GI cancers, we found that inadequate emotional support was associated with lower physical and mental HRQoL as well as increased odds of depression. Importantly, the differences in physical and mental HRQoL are large enough to meet the threshold for the minimal important change (*β* >2) of a PROMIS® instrument, meaning that it is a clinically significant change.^36^ Patients with inadequate emotional support were more likely to be from a non‐White race, disabled, or not currently married (single, divorced, widowed, etc.). Notably, inadequate emotional support was not associated with worse overall survival.

Much of the previous work on support in patients with cancer has been focused on social support broadly (encompassing emotional/informational, tangible, affectionate, and positive social‐interaction subdomains as defined by the MOS) rather than focusing on emotional support specifically. Given that emotional support is one component of social support, studies that assess social support broadly are relevant to the findings of this study. However, when there are discrepancies in these findings, it is yet unknown whether these discrepancies can be attributed to the mediating effects of other domains of social support or whether there is a true discrepancy in findings.

One study in patients with breast cancer of all ages suggested that social support, in general, was associated with improved physical and mental HRQoL. Notably, they reported that the emotional support sub‐domain was more strongly linked to improved HRQoL following diagnosis than other domains of social support.^8^ These findings contributed to our initial interest in investigating this question and the current study suggests that emotional support is, in fact, directly associated with improved physical and mental HRQoL (although in a different population, older patients with GI cancer). Another study of older adults with lung and GI cancers found that among many demographic and clinical variables (including age, sex, education, marital status, number of children, type of cancer, and monthly income) social support was the only variable associated with improvements in “vitality”, “emotional well‐being”, and “general health” subcomponents of HRQoL. One possible explanation of the uniqueness of social support in effecting “vitality”, “emotional well‐being”, and “general health” is through the mediating role of emotional support. Further research is necessary to examine a direct link between emotional support and these subcomponents which would likely require a larger HRQoL instrument than the short form included in this study.

Another previous study measuring unmet social support needs among older Medicare beneficiaries with a variety of cancer types indicated that as unmet social support needs increased, mental HRQoL scores decreased. However, there was no evidence of a difference in physical HRQoL scores, which is contrary to the current results.^13^ One potential explanation is the difference in study populations. The prior study included all cancer types and a higher percentage of Stage I and II disease. Therefore, it is possible that current results are reflective of patients with low physical HRQoL due to greater disease burden.

In addition to an association between emotional support and HRQoL, the current study suggests that inadequate emotional support is associated with depression. These findings are congruent with several prior studies suggesting that patients with depression scored *lower* on measures of social or emotional support.^9^, ^37^, ^38^ Interestingly, one prospective study followed patients with cancer of all ages with baseline depression and anxiety for 12 months and found that those with better social support had greater improvements in their mental health condition compared to those with lower social support.^9^ It is notable, however, that contrary to current results many prior studies reported an association between lower social support and *anxiety* as well as depression. This is certainly surprising as it suggests that having adequate emotional support may not decrease levels of anxiety as one might expect.

Our study did not find an association between adequate emotional support and overall survival. Yet, prior literature supports an association between larger social support networks and decreased mortality. Social networks and social support are separate but related concepts. Social networks are “the web of social relationships that surround an individual” whereas social support is one way that social networks exert their influence on cancer outcomes such as survival and quality of life.^39^, ^40^ A large meta‐analysis compiled and analyzed 87 prior studies on the association between social support and mortality among cancer patients of all ages and found “clear and unequivocal evidence for the association of social network with longevity in cancer patients”.^41^ There are a few possible explanations for the discrepancy in these findings. First, perhaps survival benefits from a larger social network can be more easily attributed to other aspects of social support including tangible support, affectionate support, and positive social interaction than the emotional support component. Second, the authors do concede that older adults “invest more energy in maintaining close and supportive social ties than in maintaining large social networks” and that therefore older adults “may be at higher risk for not having a sufficient number of social ties than for lacking emotional support.” A more recent systematic review examined the relationship between social support (defined by marital status or self‐reported assessment) and survival in colorectal cancer patients. It found that patients with social support were generally diagnosed at an earlier stage and had better survival than patients without social support.^42^ The authors speculate in the discussion that the association between higher social support and improved survival may be attributed to friends and families encouraging their loved ones to undergo colorectal cancer screening. If social support primarily affects survival by increasing the rates of screening, then we would not necessarily expect an association between emotional support and survival. Additionally, the current study did not focus on colorectal cancer alone but included other GI cancers as well, most notably pancreatic cancer which is often diagnosed at a later stage. One prior narrative review on social networks and mortality suggested that the magnitude to which social networks affect mortality is associated with cancer stage.^40^ Taken together, our focus on emotional support rather than social support broadly and our inclusion of pancreatic cancers at advanced stages may explain the lack of an association between emotional support and mortality.

Our study is not without limitations. This study was conducted in one medical center in the Southeastern United States and therefore the results may not be generalizable to other populations. Second, >80% of patients reported adequate emotional support for each question, meaning that the frequency of inadequate emotional support was somewhat low. This is, however, consistent with previous studies, but limits the ability to determine the specific source of emotional support deficiencies among older patients with cancer.^37^ Third, HRQoL and emotional support were measured at one point in time, so we are unable to establish causality. Fourth, much of the study data was patient reported which introduces information bias (e.g., perhaps patients with depression were less likely to recall instances of emotional support). Furthermore, we relied on patient reported survey measures of anxiety and depression (i.e., PROMIS Short Forms for Anxiety and Depression) which may not universally correlate with a clinical diagnosis of anxiety or depression that would be made by a medical provider. Finally, our study did not measure the difference between received and perceived emotional support. Prior research has shown demonstrable differences between the amount of support that a patient *receives* and the amount of support that they *perceive*.^43^

In this study of emotional support in older adults with GI cancers, adequate emotional support was associated with improved HRQoL and decreased odds of depression, but there was no evidence of an effect on anxiety or survival. Further work is necessary to investigate whether adequate emotional support is related to improvements in HRQoL and mental health in other cancers as well in older adults. Additionally, future studies that address the effect of social support should fractionate the contribution of its various components (emotional, informational, tangible, etc.) to better enable targeted interventions.

## AUTHOR CONTRIBUTIONS

**Daniel Clausing:** Conceptualization (lead); data curation (equal); formal analysis (supporting); investigation (lead); writing – original draft (lead); writing – review and editing (lead). **Mackenzie E Fowler:** Formal analysis (lead); methodology (lead); software (lead); visualization (lead). **Christian Harmon:** Data curation (lead); software (lead); writing – review and editing (supporting). **Abigail Tucker:** Data curation (supporting); formal analysis (supporting). **Darryl Outlaw:** Project administration (supporting); resources (supporting); supervision (supporting); writing – review and editing (supporting). **Mehmet Akce:** Conceptualization (supporting); investigation (supporting); project administration (supporting); resources (supporting). **Bassel El‐Rayes:** Funding acquisition (supporting); project administration (supporting); resources (equal); writing – review and editing (supporting). **Smith Giri:** Formal analysis (supporting); methodology (supporting); resources (equal); writing – review and editing (supporting). **Grant R. Williams:** Conceptualization (lead); funding acquisition (lead); project administration (lead).

## FUNDING INFORMATION

Supported in part by the National Cancer Institute of the National Institutes of Health (K08CA234225) and the Doris Duke Charitable Foundation CARES program at UAB. The content is solely the responsibility of the authors and does not necessarily represent the official views of the National Institutes of Health.

## Data Availability

The data that support the findings of this study are available from the corresponding author upon reasonable request
